# Vision for *Mediators of Inflammation*

**DOI:** 10.1155/2020/9202849

**Published:** 2020-02-07

**Authors:** Anshu Agrawal

**Affiliations:** Division of Basic and Clinical Immunology, Department of Medicine, University of California, Irvine, Irvine, CA 92697, USA

I am honored to be invited to take on the role of Chief Editor for *Mediators of Inflammation*. Firstly, I would like to thank the editors and staff, whose sustained efforts and support have established *Mediators of Inflammation* as a quality journal that offers a high standard of service to both its readership and its authors. I intend to build upon this good work and to use this solid position to further the reputation and impact of the journal going forward.

The highest priority for a scientific journal is to attract the best scientific authors and papers to secure its position at the forefront of academic research. *Mediators of Inflammation* has a broad scope, including the relationship between disease and inflammation, mechanisms that lead to chronic inflammation, and mechanisms that play a role in the resolution of inflammation. Emerging themes in the field are tissue-specific inflammation, tissue repair, and regeneration after an infection or injury. Further broad areas of public appeal are the effect of both macro- and micronutrients on inflammation. To realize the goal of attracting the best research, I wish to encourage the submission of a series of articles focused on inflammation in various tissues.

The primary task of an editor is to ensure that all submitted articles receive expert, courteous, and timely reviews and that editorial decisions are consistent and transparent. Moving forward, *Mediators of Inflammation* will look for results that are of significant interest to a wide audience. I am keen to work on enhancing our guidelines for transparency, while also maintaining the highest scientific standards. Accompanying these higher standards, we will also work towards reducing the time it takes to review to better serve our authors. I support the move towards transparent peer review and will consider how the journal can drive research to be more open.

I greatly look forward to working with the *Mediators of Inflammation* community on the continuing success of the journal and hope that together we will be able to make the journal a platform for the best of inflammation research in the coming years.

*Anshu Agrawal*

